# Phenotypic characteristics and rehabilitation effect of children with regressive autism spectrum disorder: a prospective cohort study

**DOI:** 10.1186/s12888-024-05955-1

**Published:** 2024-07-19

**Authors:** Chaoqun Hu, Ting Yang, Jie Chen, Ying Dai, Hua Wei, Qionghui Wu, Hongyu Chen, Dan Long, Yuru Feng, Qiuhong Wei, Qian Zhang, Li Chen, Tingyu Li

**Affiliations:** Growth, Development and Mental Health Center of Children and Adolescents, Chongqing Key Laboratory of Child Neurodevelopment and Cognitive Disorders, National Clinical Research Center for Child Health and Disorders, Ministry of Education Key Laboratory of Child Development and Disorders, Children’s Hospital of Chongqing Medical University, Chongqing, China

**Keywords:** Regressive autism spectrum disorder, Core symptoms, Rehabilitation effect, Phenotypic characteristics

## Abstract

**Background:**

In this prospective cohort study, we determined the phenotypic characteristics of children with regressive autism spectrum disorder (ASD) and explored the effects of rehabilitation.

**Methods:**

We recruited 370 children with ASD aged 1.5–7 years. Based on the Regression Supplement Form, the children were assigned to two groups: regressive and non-regressive. The core symptoms and neurodevelopmental levels of ASD were assessed before and after 1 year of behavioral intervention using the Autism Diagnostic Observation Schedule (ADOS), Social Response Scale (SRS), Children Autism Rating Scale (CARS), and Gesell Developmental Scale (GDS).

**Results:**

Among the 370 children with ASD, 28.38% (105/370) experienced regression. Regression was primarily observed in social communication and language skills. Children with regressive ASD exhibited higher SRS and CARS scores and lower GDS scores than those with non-regressive ASD. After 1 year of behavioral intervention, the symptom scale scores significantly decreased for all children with ASD; however, a lesser degree of improvement was observed in children with regressive ASD than in those with non-regressive ASD. In addition, the symptom scores of children with regressive ASD below 4 years old significantly decreased, whereas the scores of those over 4 years old did not significantly improve. Children with regressive ASD showed higher core symptom scores and lower neurodevelopmental levels. Nevertheless, after behavioral intervention, some symptoms exhibited significant improvements in children with regressive ASD under 4 years of age.

**Conclusion:**

Early intervention should be considered for children with ASD, particularly for those with regressive ASD.

**Supplementary Information:**

The online version contains supplementary material available at 10.1186/s12888-024-05955-1.

## Introduction

Autism spectrum disorder (ASD) encompasses a group of neurodevelopmental disorders with a complex etiology and strong clinical heterogeneity. It is characterized by different degrees of social interaction and communication disorders, and the primary clinical symptoms are repetitive stereotypic behavior and narrow interest [1]. The alarming increase in the prevalence of ASD over the last two decades poses an important public health concern [2]. A major challenge is the high heterogeneity in the etiology, clinical phenotype, and treatment of ASD [3–6]. Classifying the subtypes of different dimensions can help better understand the possible etiology of ASD.

Developmental regression was first reported in 1908 when Theodor Heller described severe regression in the adaptive function of children; he named this condition dementia infantilis [7]. Since then, several studies have focused on the issue of developmental regression in children with ASD [8–10], including our previous multicenter study [11]. The Autism Diagnostic Interview-Revised (ADI-R) defines developmental regression in ASD as the appearance of developmental skills such as language, social, motor, and other skills for > 3 months at an appropriate age, followed by a significant or complete disappearance of one or more skills for at least 3 months [12, 13]. Meta-analyses have revealed that the overall incidence of regressive ASD is approximately 30% [14, 15]. Consistent with the findings of previous studies [16–20], in our previous multicenter study, we observed that the incidence of regression was 13.44%; the types of regression primarily included language, social, motor skill, and mixed regression [11]. Furthermore, we noted that children with regressive ASD had poorer neurodevelopmental levels and more severe core symptoms than those with non-regressive ASD. However, similar to most current literature on regressive ASD, the clinical manifestations of regression were not fully observed in our previous multicenter study, with a focus on only language, social, and motor skill regression [11]. Furthermore, past studies on regressive ASD have primarily focused on its morbidity, clinical manifestations, and etiology, with only a few studies investigating regressive ASD prognosis. Only one small sample study on the follow-up of regressive ASD symptoms is available; it included 19 children with regressive ASD and 33 children with non-regressive ASD. At 1-year follow-up after enrollment, children with regressive ASD exhibited more severe ASD symptoms and lower neurodevelopmental levels than those with non-regressive ASD [21].

Therefore, additional studies with larger sample sizes are warranted to further investigate regressive ASD prognosis. In the present study, we employed a combination of the ADI-R and Regression Supplement Form for the multidimensional standardized assessment of regression to achieve more accurate prevalence rates. Furthermore, we established a cohort of young individuals with ASD to determine the effects of rehabilitation across different ages and regressive phenotypes.

## Methods

### Participants

Between September 2019 and October 2023, children with ASD aged 1.5–7 years were recruited from the outpatient department of the Children’s Hospital of Chongqing Medical University and cooperating special education institutions. This clinical research project was registered in the Chinese Clinical Trial Registration Center (registration number: ChiCTR2000031194, retrospective registered on 23/03/2020). Furthermore, it was approved by the Medical Ethics Committee of the Children’s Hospital of Chongqing Medical University (Ethics approval number: 121-1/2018). Before starting the study, all parents or guardians of the children provided written informed consent. In total, 398 children with ASD were recruited; 370 children who completed the regression questionnaire were included. After diagnosis, all children with ASD received a Comprehensive Treatment Model (CTMs) behavioral interventions based on the application of Applied Behavior Analysis (ABA). The characteristics of this model are as follows: (a) High intensity, with 20–40 h per week of intervention. (b) Individualized to meet the specific needs of each child. (c) Simultaneous targeting of multiple skills, rather than focusing solely on one specific skill (e.g., joint attention). (d) Utilization of various behavioral analysis methods. (e) Initially implemented in a one-on-one format, gradually transitioning to small group activities and eventually transferring skills to natural environments. Throughout the intervention, the rehabilitation training staff of the collaborating special education institutions diligently recorded the content and duration of the training sessions, while also actively participating in regular training sessions provided by our center. The symptom and developmental scale scores of children with ASD in the regressive and non-regressive groups were compared before and after the intervention to assess the impact of the targeted teaching methods and the interventions on their progress. Out of the 370 children with ASD participating in this study, 176 of them underwent reassessment after receiving behavioral interventions.

### Selection criteria

The inclusion criteria for ASD were as follows: ASD was diagnosed based on structured interviews conducted by experienced developmental-behavioral pediatricians according to the diagnostic criteria for ASD in the Diagnosis and Statistical Manual of Mental Disorders-fifth edition [22]. The diagnosis of each child was verified using the Autism Diagnostic Observation Schedule (ADOS) [23] and a Children Autism Rating Scale (CARS) score of at least 30 [24]. The exclusion criteria for ASD were as follows: (1) children with other developmental disorders or neurological or psychiatric diseases, including cerebral palsy or chronic epilepsy; (2) those with severe visual or hearing loss or other sensory impairments; (3) those with a history of serious physical disease, severe head trauma, or other conditions affecting overall growth and development; (4) those who failed to complete the regression questionnaire or obtain informed consent from parents.

The inclusion criteria for regression were as follows: (1) the occurrence of developmental milestones such as social, motor, and other skills in children with ASD for at least 3 months at the appropriate age, followed by significant or complete loss of one or more skills for > 3 months; (2) language regression was defined as the loss of some or all language skills for > 3 months after acquiring at least five words and using them for at least 3 months; (3) the first onset of regressive symptoms was before the age of 3 years. The exclusion criteria for regression were as follows: (1) loss of skills that were not entirely mastered (i.e., < 3 months after their emergence) and (2) duration of skill loss of < 3 months, suggesting a short-time loss that recovered within 3 months.

### Scales and questionnaires

#### General questionnaire

A general questionnaire was utilized to collect the basic information on the recruited children. Furthermore, the recruited participants were asked to complete the Regression Supplement Form [25], a tool used to assess developmental regression in children with ASD. This form comprises 19 items: 18 compulsory items and 1 optional item. Based on the regressive items in the ADI-R and Regression Supplement Form, the children with ASD were assigned to two groups: regressive and non-regressive.

#### Social responsiveness scale (SRS)

Based on the child’s behavior, the caregivers completed the SRS. The SRS comprises 65 items and 5 sub-items: social awareness, social cognition, social communication, motivation, and autistic mannerisms. A score of 60–75 indicates mild-to-moderate ASD, whereas a score of ≥ 76 indicates severe ASD [26]. In this study, this scale was only used in children with ASD who were aged > 4 years.

#### CARS

CARS was utilized to determine autism severity. It comprises 15 items, each classified into 1–4 severity levels, with a total score of 15–60. A total score of 30–36 indicates mild-to-moderate autism, whereas a score > 36 indicates severe autism [24, 27]. In this study, this scale was only used in children with ASD who were aged 2–6 years.

#### ADOS

ADOS is a semi-structured, standardized diagnostic tool for ASD [23]. Each module covers four areas: social interaction, communication, play, and imagination. The diagnostic score differs based on the module selected and the age of the individuals suspected of having ASD. The results are classified into three categories: typical autism, ASD, and non-ASD.

#### Gesell developmental scale (GDS)

GDS is a tool that assesses development; it is suitable for children aged 0–6 years. It comprises five items: adaptive behavior, gross motor, fine motor, language, and personal social behavior [28, 29]. The developmental quotient is classified into three types: normal (≥ 85), borderline (76–84), or delayed (≤ 75).

#### Infant-junior Middle School Student’s ability of Social Life Scale (SM)

The SM is a scale that assesses adaptive ability; it is suitable for children aged 6 months–14 years; it comprises 132 items [30]. A standard score of ≦ 8 indicates deficiencies, 9 indicates borderline deficiencies, 10 indicates normal, and ≧ 11 indicates excellent.

#### Statistical analysis

SPSS software (version 26.0, Inc., USA) was used to perform statistical analysis. Data normality was assessed using the Shapiro–Wilk or Kolmogorov–Smirnov test. Continuous variables were expressed as the mean ± standard deviation (variables match the normal distribution) or medians (interquartile ranges) (variables do not match the positive distribution). Categorical variables were presented as n (%). The chi-squared test, Mann–Whitney test, and independent samples t-test were used to determine differences among groups. Furthermore, to compare the scale scores between children with regressive and non-regressive ASD, multiple linear regression models, adjusted for age and sex, were used. Finally, the changes in the scale scores of children with ASD before and after intervention were determined using paired sample t-tests (changes match the normal distribution) or paired sample Wilcoxon signed-rank tests (changes do not match the normal distribution). *P* < 0.05 was considered to indicate statistical significance.

## Results

### Demographic characteristics

**Fig. 1:**
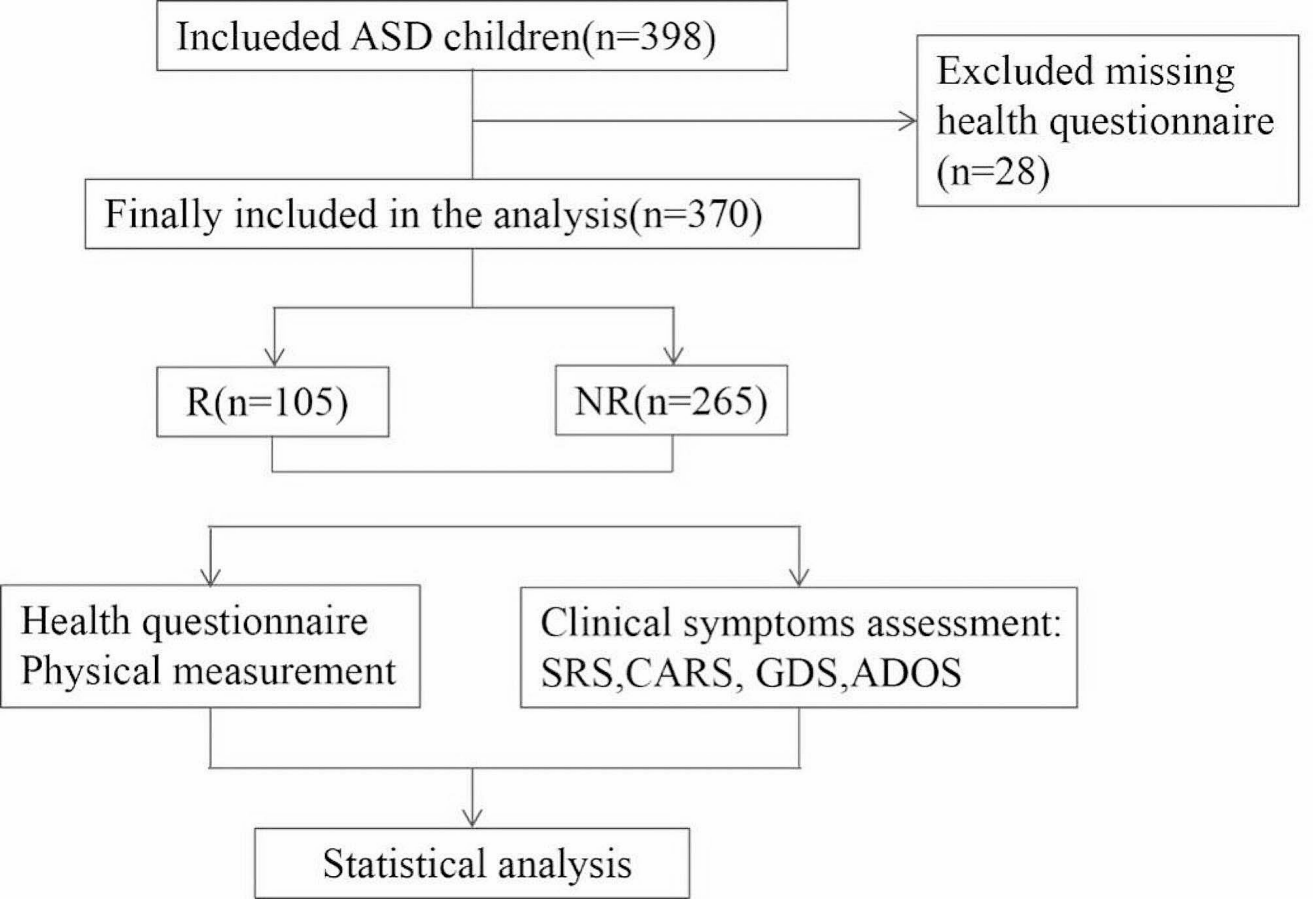
Flowchart depicting the inclusion of the study participants. R, regressive group; NR, non-regressive group

A total of 370 children with ASD whose ages ranged from 1.5 to 7 years, with an average age of 3.69 ± 1.00 years, were enrolled in the present study (Fig. 1). Among them, 287 were males and 83 were females, yielding a male: female ratio of 3.46:1. Out of the 370 patients, 105 (28.38%) were classified as having regressive ASD, whereas the remaining 265 (71.62%) were classified as having non-regressive ASD. The average age of the onset of regression was 22.34 ± 5.67 months. No significant differences were observed between the two groups regarding age (*P* = 0.054), gender (*P* = 0.689), ethnicity (*P* = 0.208), residence (*P* = 0.150), mother’s education level (*P* = 0.263), and annual family income (*P* = 0.130) (Table 1).

**Table 1:** Comparison of basic demographic characteristics between R-ASD and NR-ASD

Item	R (*n* = 105)	NR (*n* = 265)	Test	*P*
**Average age(years)**	3.73 ± 0.89	3.68 ± 1.04	*T* = -0.449	0.054
**Gender**, **n(%)**				
Male Female	80 (76.19) 25 (23.81)	207 (78.11) 58 (21.89)	x^*2*^ = 0.160	0.689
**Ethnicity**, **n (%)**				
Han Others Miss	85 (80.95) 15 (14.29) 5 (4.76)	213 (80.38) 27 (10.19) 25 (9.43)	x^*2*^ = 3.140	0.208
**Residence**, **n(%)**				
Urban Rural Miss	86 (81.90) 15 (14.29) 4 (3.81)	211 (79.62) 29 (10.94) 25 (9.43)	x^*2*^ = 3.790	0.150
**Maternal education level**, **n(%)**				
Junior high school degree or below Senior high school degree College degree or above Miss	23 (21.90) 23 (21.90) 54 (51.43) 5 (4.76)	42 (15.85) 63 (23.77) 134 (50.57) 26 (9.81)	x^*2*^ = 3.982	0.263
**Annual family income**, **RMB**, **n**(**%**)				
< 50,000	16 (15.24)	45 (16.98)		
50,000–100,000	42 (40.00)	107 (40.38)	x^*2*^ = 5.642	0.130
> 100,000	41 (39.05)	79 (29.81)		
Miss	6 (5.71)	34 (12.83)		

*Abbreviations* R-regressive group; NR-Non-regressive group

Data is shown as the number (percentage) or mean ± SD. The chi-square test and two-sample independent T-test were used in the analysis

### General description of developmental regression

Developmental regression in children with regressive ASD primarily manifested in orienting to name and direct gazing, as well as multiple social aspects and language skills. Specifically, 85 (80.95%) patients showed regression in orienting to name, whereas 84 (80.00%) showed regression in direct gazing. Additionally, language regression was prevalent, with 62 (59.05%) patients experiencing regression in speaking phrases composed of at least five words and 34 (32.38%) patients experiencing regression in speaking phrases composed of two–three words. Moreover, other social regressions included 59 (56.19%) patients of regression in social games/play, 44 (41.90%) of interest/watches in children, 32 (30.48%) of social smiling, and 21 (20.00%) of nonverbal communication. Regression in the spontaneous imitation of actions was observed in 22 (20.95%) patients, whereas regression in exhibiting gross motor skills was observed in 8 (7.62%). Additionally, minor regressions were observed in showing and paying direct attention to objects, asking for help, being quieter than other children, imaginative/pretend playing, and offering to share (Table 2)

**Table 2:** Detailed description of the regressive skills

Regressive skills	Frequency/Percentage(*n* = 105)
Orient to name	85 (80.95%)
Direct gaze	84 (80.00%)
At least five single words	62 (59.05%)
Social games/play	59 (56.19%)
Interest in/watches children	44 (41.90%)
Two- to three-word phrases	34 (32.38%)
Social smiling	32 (30.48%)
Spontaneous imitation of actions	22 (20.95%)
Nonverbal communicative gestures	21 (20.00%)
Motor Skills	8 (7.62%)
Show and direct attention to objects	6 (5.71%)
Asks for help	5 (4.76%)
Being quieter than other children	4 (3.81%)
Eye gaze/vocalization to communicate	3 (2.86%)
Offer to share	2 (1.90%)
Share the enjoyment with others	2 (1.90%)
Imaginative/pretend play	2 (1.90%)
Appropriate response to social overtures from adults	0

### Associations between regressive phenotypes and symptom scale scores

Compared with the non-regressive group, the regressive group displayed significantly higher scores regarding the SRS total score (β = 9.461, *P* < 0.001), social perception (β = 0.920, *P* = 0.008), social cognition (β = s1.343, *P* = 0.009), social communication (β = 2.996, *P* = 0.002), social motivation (β = 1.889, *P* = 0.004), autistic behavior pattern (β = 2.313, *P* = 0.002), CARS total score (β = 2.911, *P* < 0.001), ADOS comparison score (β = 1.302, *P* = 0.001), SA (β = 3.135, *P* < 0.001), and RRB (β = 0.671, *P* = 0.040) (Table 3). These findings suggested that children with regressive ASD exhibited more severe core symptoms compared with those with non-regressive ASD.

**Table 3:** Comparison of symptom scale scores between R-ASD and NR-ASD

Item	R (*n* = 105)	NR (*n* = 265)	Β (95%CI)	*P*
SRS				
Social awareness	11.95 ± 2.95	11.00 (10.00–13.00)	0.920 (0.241,1.599)	0.008
Social cognition	18.21 ± 4.49	17.00 (15.00–20.00)	1.343 (0.342,2.344)	0.009
Social communication	34.81 ± 9.68	32.37 ± 7.41	2.996 (1.138,4.853)	0.002
Social motivation	14.00 (12.00–20.00)	14.00 (11.00–19.00)	1.889 (0.599,3.179)	0.004
Autistic mannerisms	14.57 ± 7.60	12.00 (9.00–16.00)	2.313 (0.875,3.752)	0.002
SRS total score	95.57 ± 26.37	88.29 ± 20.19	9.461 (4.329,14.593)	< 0.001
**CARS**				
CARS total score	38.50 (33.75-42.00)	34.00 (31.00–39.00)	2.911 (1.284,4.538)	< 0.001
**ADOS**				
ADOS comparison score	7.00 (6.00–8.00)	6.00 (0.00–7.00)	1.302 (0.573,2.032)	0.001
SA	16.00 (12.00–18.00)	13.00 (8.00–17.00)	3.135 (1.707,4.563)	< 0.001
RBB	2.00 (1.00-4.75)	2.00 (1.00–3.00)	0.671 (0.029,1.312)	0.040

*Abbreviations* SA-social affect; RRB-restrictive and repetitive behaviors

Multivariate linear regression was used for the adjusted model, adjusted for age and gender; *β*(95%CI), regression coefficient (95% confidence interval). Data is presented as the mean ± SD or Median (IQR)

### Associations between regressive phenotypes and developmental scale scores

Compared with the non-regressive group, the regressive group showed significantly lower scores for GDS adaptive behavior (β = −5.879, *P* = 0.006), gross movement (β = −5.202, *P* = 0.010), fine movement (β = −5.969, *P* = 0.010), language (β = −8.208, *P* = 0.001), and personal socialization (β = −7.869, *P* < 0.001). Additionally, the SM standard scores in the regression group were significantly lower than those in the non-regressive group (β = −0.283, *P* = 0.017) (Table 4). These results indicated that the neurodevelopmental level and adaptive ability of children with regressive ASD were lower than those of children with non-regressive ASD.

**Table 4:** Comparison of the developmental scale scores between R-ASD and NR-ASD

Item	R (*n* = 105)	NR (*n* = 265)	Β (95%CI)	*P*
GDS				
Adaptive behavior	64.60 ± 14.89	65.72 ± 15.94	-5.879 (-10.093,-1.664)	0.006
Gross motor	76.60 ± 15.16	76.14 ± 16.24	-5.202 (-9.143,-1.260)	0.010
Fine motor	73.43 ± 16.22	76.00 ± 17.21	-5.969 (-10.517,-1.421)	0.010
Language	45.57 ± 19.49	54.00 (36.00–62.00)	-8.208 (-13.021,-3.396)	0.001
Personal social behavior	55.38 ± 13.05	58.60 ± 14.66	-7.869 (-11.924,-3.814)	< 0.001
DQ	62.00 (56.00-71.50)	65.37 ± 14.43	-3.592 (-7.372,0.187)	0.062
**SM**	9.00 (8.00–9.00)	9.00 (8.00–9.00)	-0.283 (-0.514,-0.051)	0.017

Multivariate linear regression was performed for the adjusted model, adjusted for age and gender; *β*(95%CI), regression coefficient (95% confidence interval). Data is presented as the mean ± SD or Median (IQR)

### Comparison of the symptom and developmental scale scores of children with ASD in the regressive and non-regressive groups before and after the intervention

Out of the 370 children with ASD enrolled in the present study, 176 were followed up after a year of behavioral intervention at special education institutions. Among them, 120 (68.18%) were in the non-regressive group, and the remaining 56 (31.82%) were in the regressive group. A significant decrease was observed for CARS total score (Z = − 4.760, *P* < 0.001), SRS total score (Z = − 3.791, *P* < 0.001), social awareness (Z = − 3.181, *P* = 0.001), social cognition (Z = − 2.756, *P* = 0.006), social communication (t = 4.360, *P* < 0.001), and social motivation (t = 4.189, *P* < 0.001) in the non-regressive group. Conversely, a significant decrease was observed for only CARS total score (t = 2.886, *P* = 0.006), social communication (t = 2.140, *P* = 0.038), and social motivation (t = 2.247, *P* = 0.029) in the regressive group (Table 5). These results indicated that, after a year of the behavioral intervention, the core autism symptoms improved more significantly in the non-regressive group than in the regressive group. The regressive phenotypes probably significantly affected the effectiveness of behavioral intervention in children with ASD.

**Table 5:** Comparison of symptom scale and developmental scale scores of R-ASD and NR-ASD before and after intervention

Item	NR (n1 = 120)				*R* (n2 = 56)			
	Basal time	12 months	Test	*P*	Basal time	12 months	Test	*P*
CARS total score	34.00 (31.00–40.00)	31.00 (29.00–36.00)	*Z*=-4.760	< 0.001	37.90 ± 6.17	35.95 ± 6.15	*t* = 2.886	0.006
ADOS total score	15.00 (12.00-19.75)	14.00 (11.00–19.00)	*t* = 1.578	0.118	17.77 ± 4.63	17.00 ± 5.88	*Z*=-1.474	0.141
ADOS comparison score	6.00 (5.00–7.00)	7.00 (6.00–8.00)	*Z*=-1.795	0.073	7.00 (6.00–8.00)	7.32 ± 1.91	*t*=-0.764	0.449
SA	12.79 ± 4.52	12.31 ± 4.15	*Z*=-1.704	0.088	16.00 (14.00–18.00)	15.00 (11.00–17.00)	*Z*=-1.785	0.074
RRB	2.00 (1.00–3.00)	2.00 (1.00–3.00)	*Z*=-0.345	0.730	2.00 (1.00–4.00)	2.00 (1.00–4.00)	*Z*=-0.274	0.784
SRS total score	90.29 ± 21.62	80.28 ± 26.22	*Z*=-3.791	< 0.001	99.24 ± 24.19	89.45 ± 29.93	*t* = 1.791	0.080
Social awareness	11.43 ± 2.78	10.00 (8.00–13.00)	*Z*=-3.181	0.001	11.63 ± 2.86	11.50 ± 3.06	*Z*=-0.411	0.681
Social cognition	17.00 (15.00–20.00)	16.19 ± 4.62	*Z*=-2.756	0.006	19.63 ± 5.18	17.74 ± 5.32	*t* = 1.368	0.178
Social communication	32.41 ± 7.45	28.91 ± 10.02	*t* = 4.360	< 0.001	36.03 ± 8.31	31.63 ± 11.42	*t* = 2.140	0.038
Social motivation	15.00 (11.00–20.00)	12.00 (9.00–16.00)	*t* = 4.189	< 0.001	16.58 ± 5.71	14.50 ± 5.25	*t* = 2.247	0.029
Autistic mannerisms	13.43 ± 5.78	11.85 ± 6.05	*Z*=-1.653	0.098	15.37 ± 7.37	14.50 (8.00–18.00)	*t* = 0.151	0.880
GDS DQ	70.97 ± 14.58	69.98 ± 17.20	*t* = 1.533	0.129	66.40 ± 13.56	61.32 ± 17.61	*Z*=-0.959	0.337
Adaptive behavior	69.20 ± 16.58	70.76 ± 20.37	*t*=-0.477	0.634	62.58 ± 13.95	61.21 ± 19.73	*Z*=-1.341	0.180
Gross motor	81.39 ± 18.10	77.41 ± 15.59	*t* = 2.439	0.017	73.00 ± 13.48	70.18 ± 12.98	*t* = 0.801	0.427
Fine motor	79.57 ± 17.93	74.89 ± 16.18	*t* = 3.126	0.002	71.53 ± 14.86	68.00 (59.25–77.75)	*t* = 1.161	0.252
Language	54.00 (41.00–68.00)	59.75 ± 23.60	*t*=-1.956	0.053	46.74 ± 19.38	42.00 (34.50-57.75)	*t*=-1.030	0.309
Personal social behavior	62.85 ± 17.20	66.92 ± 20.85	*Z*=-1.650	0.603	54.34 ± 13.23	58.00 ± 23.03	*t*=-1.059	0.295
SM	9.00 (9.00–10.00)	9.00 (8.00–10.00)	*Z*=-0.014	0.989	9.00 (8.00–9.00)	9.00 (8.00–10.00)	*Z = 0.000*	1.000

Data are presented as the mean ± SD or median (IQR). Paired sample t-test or paired samples Wilcoxon signed rank-test was employed for analysis

### Associations between age and intervention effectiveness

A significant decrease was observed in the CARS total score (Z = − 5.405, *P* < 0.001), ADOS comparison score (Z = − 2.218, *P* = 0.027), SA (Z = − 2.005, *P* = 0.045), SRS total score (t = 4.321, *P* < 0.001), social cognition (Z = − 2.253, *P* = 0.024), social communication (t = 3.944, *P* < 0.001), and social motivation (t = 4.316, *P* < 0.001) for children aged < 4 years in the non-regressive group. In addition, a significant decrease was observed for CARS total score (t = 3.227, *P* = 0.003), ADOS total score (t = 2.060, *P* = 0.049), SRS total score (t = 2.274, *P* = 0.031), social communication (t = 2.220, *P* = 0.035), and social motivation (t = 2.416, *P* = 0.023) in the regressive group (Tables 6 and 7). For the non-regressive children, the language scores of patients aged less than 4 years depicted a significant improvement after behavioral intervention. However, no significant difference in the language scores was observed for patients aged > 4 years. On the other hand, for regressive children, no significant improvement was noted in the language scores in both the age groups

**Table 6:** Effect of age on the effects of behavioral interventions for NR-ASD

Item	< 4 years (*n* = 83)				≥ 4 years (*n* = 37)			
	Basal time	12 months	Test	*P*	Basal time	12 months	Test	*P*
CARS total score	35.80 ± 6.02	31.00 (28.00–36.00)	*Z*=-5.405	< 0.001	34.21 ± 5.94	32.00 (30.50–35.50)	*t*=-0.421	0.678
ADOS total score	14.00 (11.00–19.00)	13.00 (11.00–18.00)	*t* = 1.917	0.060	15.44 ± 5.00	15.44 ± 5.17	*t* = 0.000	1.000
ADOS comparison score	6.00 (4.00–7.00)	6.00 (6.00–8.00)	*Z*=-2.218	0.027	6.00 (6.00-8.75)	6.97 ± 1.49	*Z*=-0.611	0.541
SA	13.27 ± 4.14	12.13 ± 4.18	*Z*=-2.005	0.045	12.66 ± 4.25	12.63 ± 4.26	*t* = 0.056	0.955
RRB	2.00 (1.00–2.00)	2.00 (1.00–3.00)	*Z*=-0.591	0.554	2.00 (1.00–4.00)	2.81 ± 1.82	*t*=-0.090	0.929
SRS total score	90.65 ± 20.69	80.02 ± 26.56	*t* = 4.321	< 0.001	89.38 ± 24.36	80.95 ± 25.97	*t* = 1.685	0.102
Social awareness	11.30 ± 2.52	10.00 (8.00-12.25)	*t* = 2.578	0.012	11.76 ± 3.40	10.00 (8.00-13.50)	*t* = 1.933	0.062
Social cognition	17.96 ± 3.99	16.35 ± 4.74	*Z*=-2.253	0.024	17.52 ± 5.04	15.76 ± 4.38	*t* = 1.584	0.123
Social communication	32.63 ± 7.43	28.70 ± 10.40	*t* = 3.944	< 0.001	31.86 ± 7.66	29.43 ± 9.20	*Z*=-1.501	0.133
Social motivation	15.00 (11.00–20.00)	12.00 (9.00-16.25)	*t* = 4.316	< 0.001	14.05 ± 5.14	12.14 ± 5.02	*t* = 1.167	0.252
Autistic mannerisms	13.13 ± 5.77	11.30 ± 6.14	*Z*=-1.845	0.065	14.19 ± 5.85	13.29 ± 5.70	*t* = 0.602	0.552
GDS DQ	72.00 ± 13.94	70.84 ± 15.67	*t* = 1.727	0.088	68.33 ± 16.19	67.78 ± 20.92	*t* = 0.473	0.641
Adaptive behavior	71.04 ± 15.55	70.70 ± 17.12	*t* = 0.484	0.630	64.48 ± 18.55	70.90 ± 27.55	*t*=-1.715	0.100
Gross motor	83.59 ± 16.70	78.56 ± 14.45	*t* = 2.838	0.006	75.71 ± 20.65	74.48 ± 18.26	*Z*=-0.553	0.580
Fine motor	80.85 ± 16.55	75.81 ± 13.91	*t* = 3.019	0.003	76.29 ± 21.17	72.52 ± 21.17	*Z*=-0.837	0.402
Language	55.76 ± 21.40	60.98 ± 23.17	*t*=-2.484	0.015	50.00 (38.50–68.50)	56.57 ± 24.96	*Z*=-0.818	0.413
Personal social behavior	63.17 ± 17.24	67.94 ± 20.52	*Z*=-1.668	0.095	62.05 ± 17.51	64.29 ± 21.97	*t*=-0.667	0.512
SM	9.00 (9.00–10.00)	9.00 (9.00–10.00)	Z=-0.670	0.503	9.00 (8.00–10.00)	9.00 (8.00–10.00)	Z=-0.775	0.438

Data is presented as the mean ± SD or median (IQR). Paired sample t-test or paired samples Wilcoxon-signed rank-test was used for analysis

**Table 7:** Effect of age on the effects of behavioral interventions for R-ASD

Item	< 4 years (*n* = 35)				≥ 4 years (*n* = 21)			
	Basal time	12 months	Test	*P*	Basal time	12 months	Test	*P*
CARS total score	38.96 ± 6.46	36.36 ± 5.61	*t* = 3.227	0.003	36.44 ± 5.61	35.38 ± 6.98	*t* = 0.913	0.373
ADOS total score	19.00 (17.00–21.00)	16.75 ± 5.13	*t* = 2.060	0.049	16.89 ± 4.89	17.05 ± 7.25	*t*=-0.130	0.898
ADOS comparison score	7.00 (6.00-8.75)	7.39 ± 1.71	*Z*=-0.229	0.819	7.05 ± 1.43	7.00 (6.00–9.00)	*t*=-0.364	0.720
SA	16.00 (15.00–18.00)	14.50 ± 3.72	*t* = 1.979	0.058	14.47 ± 3.60	13.79 ± 4.66	*t* = 0.853	0.405
RRB	2.57 ± 1.87	1.50 (1.00–4.00)	*t* = 0.911	0.370	2.00 (1.00–4.00)	2.00(1.00–5.00)	*t*=-1.319	0.187
SRS total score	98.55 ± 27.41	87.82 ± 32.17	*t* = 2.274	0.031	100.19 ± 19.74	91.69 ± 27.40	*t* = 0.378	0.709
Social awareness	11.64 ± 3.16	11.27 ± 3.48	*t* = 0.775	0.445	11.63 ± 2.50	11.81 ± 2.46	*t*=-1.759	0.094
Social cognition	19.00 ± 5.14	17.73 ± 5.71	*t* = 1.012	0.321	20.50 ± 5.28	17.75 ± 4.91	*t* = 0.899	0.380
Social communication	35.82 ± 9.27	31.05 ± 12.59	*t* = 2.220	0.035	36.31 ± 7.07	32.44 ± 9.93	*t* = 0.770	0.451
Social motivation	16.91 ± 6.06	13.86 ± 4.49	*t* = 2.416	0.023	16.13 ± 5.35	15.38 ± 6.21	*t* = 0.585	0.565
Autistic mannerisms	15.18 ± 8.69	13.91 ± 8.18	*t* = 0.599	0.555	15.00 (12.25–16.75)	14.31 ± 5.91	*t*=-0.246	0.808
GDS DQ	69.14 ± 13.61	58.50 (47.8–68.5)	*Z*=-1.187	0.235	62.63 ± 12.96	59.80 ± 13.87	*Z*=-0.213	0.831
Adaptive behavior	64.64 ± 14.30	57.00 (49.75–64.25)	*t* = 1.547	0.135	59.75 ± 13.37	60.75 ± 16.83	*t*=-0.576	0.572
Gross motor	75.00 ± 14.20	72.05 ± 12.72	*t* = 0.332	0.743	70.25 ± 12.34	67.63 ± 13.31	*t* = 1.001	0.330
Fine motor	73.18 ± 15.14	68.50 ± 19.14	*t* = 1.479	0.152	69.25 ± 14.63	67.31 ± 11.51	*Z*=-0.403	0.687
Language	45.23 ± 19.26	42.50 (29.50–57.00)	*t*=-1.780	0.087	42.00 (36.25–56.75)	46.19 ± 17.58	*t* = 0.800	0.434
Personal social behavior	56.68 ± 14.57	48.00 (39.50-69.75)	*Z*=-0.877	0.381	51.13 ± 10.75	57.25 ± 19.12	*t*=-1.475	0.157
SM	9.00 (8.00-9.25)	9.00 (8.00–10.00)	Z=-0.225	0.822	9.00 (8.00–9.00)	8.63 ± 0.96	Z=-0.333	0.739

Data are presented as the mean ± SD or median (IQR). Paired sample t-test or paired samples Wilcoxon-signed rank-test was employed for analysis

## Discussion

The early identification of regressive ASD is challenging; consequently, early intervention for ASD management is difficult. Thus, understanding ASD phenotypes is crucial for clinical diagnosis and treatment. Most existing studies have included small sample sizes to investigate regressive ASD incidence in children [31, 32]. Moreover, studies on the effect of rehabilitation on children with regressive ASD are scarce. To the best of our knowledge, the present study is the largest cohort study focusing on the rehabilitation effects across different ages and regressive phenotypes, and the results highlight the need for early intervention in children with regressive ASD. We found that children with regressive ASD demonstrated higher core symptom scores and lower neurodevelopmental levels than those without. The behavioral intervention significantly improved some of these core symptoms in children under the age of 4

Herein, we have reported regressive ASD incidence of 28.38%, which was higher than that reported previously [20]. Moreover, the average age at the onset of regression was 22.34 ± 5.67 months, which is in line with previously reported results ^14^. A recent meta-analysis reported regressive ASD incidence to be approximately 30%, and the average age at the time of onset was 19.8 months [14]. Children with regressive ASD displayed more severe core symptoms of autism than do children with non-regressive ASD [16–19]. Similarly, herein, the core symptom scale scores of children with ASD were found to be significantly higher in the regressive group than in the non-regressive group, indicating that children in the regressive group showed more severe core autism symptoms. Additionally, adaptive behavior, fine motor, language, and personal social scores on the GDS and SM were significantly lower in the regressive group than in the non-regressive group, which indicated that children in the regressive ASD group showed lower neurodevelopmental levels than did those in the non-regressive ASD group. Consistent with most studies [13, 14], this study showed that children with regressive ASD primarily exhibited language and social regression. Furthermore, we found that social regression, particularly in orienting to name and direct gazing, was observed in up to 80% of the children

Past studies on categorizing the ASD rehabilitation effects on the basis of a larger sample size of regression phenotypes are lacking; however, the present study showed that, after 1 year of behavior training, the symptom scale scores of children with ASD in the non-regressive group decreased more significantly compared with those in the regressive group. These findings suggested that regressive phenotypes significantly affected the rehabilitation of children with ASD. In addition, because ASD diagnosis typically occurs after 4 years of age, studies evaluating the rehabilitation effect on children with ASD below 4 years of age are lacking. In addition, we observed that children with ASD over 4 years of age showed no significant differences in the rehabilitation effect between the regressive and non-regressive groups. However, for children with ASD below 4 years of age, a significant rehabilitation effect was observed in both the non-regressive group and the regressive group. Furthermore, the GDS language scores of children below 4 years of age in the non-regressive group significantly improved after the training. Conversely, children below 4 years of age and those below and over 4 years of age in the regressive group did not show any significant improvement in their GDS language scores. The ADOS scale scores were also consistent with these results, and in children below and over 4 years of age, partial scores in the non-regressive group decreased significantly after the year-long intervention, whereas those in the regressive group did not decrease. Although the results of the ADOS scores may not exhibit the same level of significance as the CARS and SRS scores, we believe that this discrepancy could be attributed to the differences in assessment results due to variations in the instruments utilized and the specific items they measure. CARS and SRS assess a wider range of sensory perception and social adaptation items when compared to the ADOS. Our results thus suggest that early intervention is more beneficial for improving language abilities and the overall rehabilitation effects

Consistent with our study findings, other studies have also reported that starting behavioral interventions at a younger age yields better outcomes. For example, initiating interventions at 18 months of age has shown greater therapeutic benefits compared to starting at 27 months [33]. Since the period from birth to 3 years is considered a peak period of neural plasticity and a critical stage for the establishment and consolidation of early social and communication skills, disruptions during this developmental phase can significantly interfere with subsequent successful acquisition [34, 35]. Moreover, most ASD symptoms become apparent around the age of 2. Therefore, early intervention is preferred, as it becomes increasingly challenging to repair neural circuits and behavioral expressions for achieving optimized behavioral patterns with prolonged delay. Furthermore, the “window of opportunity” theory for language suggests that children with autism who are nonverbal by the age of 4 are more likely to experience delayed language development [36, 37]. Therefore, interventions before the age of 4 may yield better therapeutic benefits in the domain of language

Based on our previous study, this large-sample cohort study further reports on rehabilitation effects on regressive autism, providing valuable insights for clinicians and parents. However, the limitations of the study must be acknowledged. It is recommended to begin interventions immediately after the diagnosis of Autism Spectrum Disorder (ASD) or even at the suspicion of ASD. Our study has a follow-up period of only one year. Some of the conclusions drawn may require further optimization. Extending the follow-up period and expanding the sample size are necessary. Therefore, our research team is currently continuing with longer-term follow-ups for further related studies

## Conclusions

The present study revealed the incidence of regression to be 28.38%, and more severe core symptoms of autism and lower levels of neurodevelopment were observed in children with regressive ASD than in those with non-regressive ASD. After a year of training, the core symptoms improved significantly in the non-regressive group and the younger age group, whereas the regressive group and the older age group showed relatively poorer outcomes in terms of improvement in the core symptoms. The regressive phenotype and the age at which training was initiated affected the effectiveness of rehabilitation. Therefore, for children with ASD, particularly those with regressive ASD, behavior training should be initiated as early as possible

### Electronic supplementary material

Below is the link to the electronic supplementary material.

Supplementary Material 1

## Data Availability

All data generated or analyzed during this study are included in this published article and its supplementary information files.

## References

[1] Lord C, Elsabbagh M, Baird G, Veenstra-Vanderweele J. Autism spectrum disorder. Lancet. 2018;392(10146):508–20. 30078460 10.1016/S0140-6736(18)31129-2 PMC7398158

[2] Zhou H, Xu X, Yan WL, Zou XB, Wu LJ, Luo XR, Li TY, Huang Y, Guan HY, Chen X, Mao M, Xia K, Zhang L, Li E, Ge X, Zhang L, Li C, Zhang X, Zhou Y, Ding D, Shih A, Fombonne E, Zheng Y, Han J, Sun Z, Jiang YH, Wang Y. LATENT-NHC study team. Prevalence of Autism Spectrum Disorder in China: a Nationwide Multi-center Population-based study among children aged 6 to 12 years. Neurosci Bull. 2020;36(9):961–71. 32607739 10.1007/s12264-020-00530-6 PMC7475160

[3] Isaksson J, Ruchkin V, Aho N, Lundin Remnelius K, Marschik PB, Bolte S. Nonshared environmental factors in the aetiology of autism and other neurodevelopmental conditions: a monozygotic co-twin control study. Mol Autism. 2022;13(1):8. 35183250 10.1186/s13229-022-00487-5 PMC8858556

[4] Schmidt RJ, Iosif AM, Angel EG, Ozonoff S. Association of Maternal Prenatal Vitamin Use with Risk for Autism Spectrum Disorder recurrence in young siblings. JAMA Psychiatry. 2019;76(4):391–8. 30810722 10.1001/jamapsychiatry.2018.3901 PMC6450282

[5] Idring S, Magnusson C, Lundberg M, Ek M, Rai D, Svensson AC, Dalman C, Karlsson H, Lee BK. Parental age and the risk of autism spectrum disorders: findings from a Swedish population-based cohort. Int J Epidemiol. 2014;43(1):107–15. 24408971 10.1093/ije/dyt262

[6] von Ehrenstein OS, Ling C, Cui X, Cockburn M, Park AS, Yu F, Wu J, Ritz B. Prenatal and infant exposure to ambient pesticides and autism spectrum disorder in children: population based case-control study. BMJ. 2019;364:l962. 30894343 10.1136/bmj.l962 PMC6425996

[7] Westphal A, Schelinski S, Volkmar F, Pelphrey K. Revisiting regression in Autism: Heller’s Dementia Infantilis includes a translation of Uber Dementia Infantilis. J Autism Dev Disord. 2013;43(2):265–71. 22677931 10.1007/s10803-012-1559-z

[8] Wolff S, Chess S, A BEHAVIOURAL STUDY OF, SCHIZOPHRENIC CHILDREN. Acta Psychiatr Scand. 1964;40(4):438–66. 14325807 10.1111/j.1600-0447.1964.tb07496.x

[9] Kurita H. Infantile autism with speech loss before the age of thirty months. J Am Acad Child Psychiatry. 1985;24(2):191–6. 3989162 10.1016/S0002-7138(09)60447-7

[10] Burd L, Fisher W, Kerbeshian J. Pervasive disintegrative disorder: are Rett syndrome and Heller dementia infantilis subtypes? Dev Med Child Neurol. 1989;31(5):609–16. 2806742 10.1111/j.1469-8749.1989.tb04046.x

[11] Hu CQ, Yang F, Yang T, Chen J, Dai Y, Jia FY, Wu LJ, Hao Y, Li L, Zhang J, Ke X, Yi M, Hong Q, Chen J, Fang S, Wang Y, Wang Q, Jin C, Li T, Chen L. A Multi-center Study on the Relationship between Developmental Regression and Disease Severity in Children with Autism Spectrum disorders. Front Psychiatry. 2022;13:796554. 35356716 10.3389/fpsyt.2022.796554 PMC8959377

[12] Lord C, Rutter M, Le Couteur A. Autism Diagnostic Interview-Revised: a revised version of a diagnostic interview for caregivers of individuals with possible pervasive developmental disorders. J Autism Dev Disord. 1994;24(5):659–85. 7814313 10.1007/BF02172145

[13] Zwaigenbaum L. Perspectives on regressive onset in autism: looking forward on looking back. Neurosci Biobehav Rev. 2019;103:399–400. 31229527 10.1016/j.neubiorev.2019.06.025

[14] Barger BD, Campbell JM, McDonough JD. Prevalence and onset of regression within Autism Spectrum disorders: a Meta-analytic review. J Autism Dev Disord. 2013;43(4):817–28. 22855372 10.1007/s10803-012-1621-x

[15] Tan C, Frewer V, Cox G, Williams K, Ure A. Prevalence and age of onset of regression in children with Autism Spectrum disorder: a systematic review and Meta-analytical update. Autism Res. 2021;14(3):582–98. 33491292 10.1002/aur.2463

[16] Boterberg S, Charman T, Marschik PB, Bolte S, Roeyers H. Regression in autism spectrum disorder: a critical overview of retrospective findings and recommendations for future research. Neurosci Biobehav Rev. 2019;102:24–55. 30917924 10.1016/j.neubiorev.2019.03.013

[17] Thompson L, Gillberg C, Landberg S, Kantzer A-K, Miniscalco C, Olsson MB, Eriksson MA, Fernell E. Autism with and without regression: a two-year prospective longitudinal study in two Population-Derived Swedish cohorts. J Autism Dev Disord. 2019;49(6):2281–90. 30734177 10.1007/s10803-018-03871-4 PMC6546868

[18] Boterberg S, Van Coster R, Roeyers H, Characteristics. Early Development and Outcome of parent-reported regression in Autism Spectrum Disorder. J Autism Dev Disord. 2019;49(11):4603–25. 31463633 10.1007/s10803-019-04183-x

[19] Bradley CC, Boan AD, Cohen AP, Charles JM, Carpenter LA. Reported history of Developmental Regression and Restricted, repetitive behaviors in children with Autism Spectrum disorders. J Dev Behav Pediatr. 2016;37(6):451–6. 27366956 10.1097/DBP.0000000000000316

[20] Gadow KD, Perlman G, Weber RJ. Parent-reported Developmental Regression in Autism: Epilepsy, IQ, Schizophrenia spectrum symptoms, and Special Education. J Autism Dev Disord. 2017;47(4):918–26. 28074354 10.1007/s10803-016-3004-1

[21] Martin-Borreguero P, Gomez-Fernandez AR, De la Torre-aguilar MJ, Gil-Campos M, Flores-Rojas K, Perez-Navero JL. Children with autism spectrum disorder and neurodevelopmental regression present a severe Pattern after a Follow-Up at 24 months. Front Psychiatry. 2021;12:644324. 33841211 10.3389/fpsyt.2021.644324 PMC8032949

[22] Maenner MJ, Rice CE, Arneson CL, Cunniff C, Schieve LA, Carpenter LA, Van Naarden Braun K, Kirby RS, Bakian AV, Durkin MS. Potential impact of DSM-5 criteria on autism spectrum disorder prevalence estimates. JAMA Psychiatry. 2014;71(3):292–300. 24452504 10.1001/jamapsychiatry.2013.3893 PMC4041577

[23] Lord C, Risi S, Lambrecht L, Cook EH Jr., Leventhal BL, DiLavore PC, Pickles A, Rutter M. The autism diagnostic observation schedule-generic: a standard measure of social and communication deficits associated with the spectrum of autism. J Autism Dev Disord. 2000;30(3):205–23. 11055457 10.1023/A:1005592401947

[24] Schopler E, Reichler RJ, DeVellis RF, Daly K. Toward objective classification of childhood autism: Childhood Autism Rating Scale (CARS). J Autism Dev Disord. 1980;10(1):91–103. 6927682 10.1007/BF02408436

[25] Goldberg WA, Osann K, Filipek PA, Laulhere T, Jarvis K, Modahl C, Flodman P, Spence MA. Language and other regression: assessment and timing. J Autism Dev Disord. 2003;33(6):607–16. 14714930 10.1023/B:JADD.0000005998.47370.ef

[26] Cen CQ, Liang YY, Chen QR, Chen KY, Deng HZ, Chen BY, Zou XB. Investigating the validation of the Chinese Mandarin version of the Social Responsiveness Scale in a Mainland China child population. BMC Psychiatry. 2017;17(1):51. 28166747 10.1186/s12888-016-1185-y PMC5292795

[27] Santos TH, Barbosa MR, Pimentel AG, Lacerda CA, Balestro JI, Amato CA, Fernandes FD. Comparing the use of the Childhood Autism Rating Scale and the Autism Behavior Checklist protocols to identify and characterize autistic individuals. J Soc Bras Fonoaudiol. 2012;24(1):104–6. 22460381 10.1590/S2179-64912012000100018

[28] Liu C, Huang L, Huang S, Wei L, Cao D, Zan G, Tan Y, Wang S, Yang M, Tian L, Tang W, He C, Shen C, Luo B, Zhu M, Liang T, Pang B, Li M, Mo Z, Yang X. Association of both prenatal and early childhood multiple metals exposure with neurodevelopment in infant: a prospective cohort study. Environ Res. 2022;205:112450. 34861232 10.1016/j.envres.2021.112450

[29] Dror R, Malinger G, Ben-Sira L, Lev D, Pick CG, Lerman-Sagie T. Developmental outcome of children with enlargement of the cisterna magna identified in utero. J Child Neurol. 2009;24(12):1486–92. 19240044 10.1177/0883073808331358

[30] Yuan J, Song J, Zhu D, Sun E, Xia L, Zhang X, Gao C, Agam G, Wang X, Blomgren K, Zhu C. Lithium treatment is safe in Children with Intellectual disability. Front Mol Neurosci. 2018;11:425. 30524233 10.3389/fnmol.2018.00425 PMC6262083

[31] Stefanatos GA. Regression in autistic spectrum disorders. Neuropsychol Rev. 2008;18(4):305–19. 18956241 10.1007/s11065-008-9073-y

[32] Ozonoff S, Li D, Deprey L, Hanzel EP, Iosif A-M. Reliability of parent recall of symptom onset and timing in autism spectrum disorder. Autism. 2018;22(7):891–6. 28903580 10.1177/1362361317710798 PMC5832544

[33] Guthrie W, Wetherby AM, Woods J, Schatschneider C, Holland RD, Morgan L, Lord CE. The earlier the better: an RCT of treatment timing effects for toddlers on the autism spectrum. Autism. 2023;15(8):13623613231159153. 10.1177/13623613231159153 PMC10502186 36922406

[34] Kolb B, Gibb R. Brain plasticity and behaviour in the developing brain. J Can Acad Child Adolesc Psychiatry. 2011;20(4):265–76. 22114608 PMC3222570

[35] Towle PO, Patrick PA, Ridgard T, Pham S, Marrus J. Is Earlier Better? The Relationship between Age When Starting Early Intervention and Outcomes for Children with Autism Spectrum Disorder: A Selective Review. Autism Res Treat. 2020; 2020:7605876. 10.1155/2020/7605876 PMC7421097 32832154

[36] Houston DM, Miyamoto RT. Effects of early auditory experience on word learning and speech perception in deaf children with cochlear implants: implications for sensitive periods of language development. Otol Neurotol. 2010;31(8):1248–53. 20818292 10.1097/MAO.0b013e3181f1cc6a PMC2996231

[37] Rutter M, Greenfeld D, Lockyer L. A five to fifteen year follow-up study of infantile psychosis. II. Social and behavioural outcome. Br J Psychiatry. 1967;113(504):1183–99. 6075452 10.1192/bjp.113.504.1183

