# Supplementary material for: Phenotypic characteristics and rehabilitation effect of children with regressive autism spectrum disorder: a prospective cohort study
Source: BMC Psychiatry. 2024 Jul 19;24:514. doi: 10.1186/s12888-024-05955-1 (PMC11264485; doi:10.1186/s12888-024-05955-1)
Supplement: Supplementary file 1 — Supplementary Material 1 [file 12888_2024_5955_MOESM1_ESM.docx]

**Supporting information**

Additional supporting information may be found in the online version of this article at the publisher's web-site:Additional Tables 1 and 2.

**Assessment of improvement in disease severity 1 year after intervention in ASD children**

Of the 370 ASD children enrolled in this study, 176 children were followed up after 1 year of behavioral intervention in special education institutions. When compared to that at the time of enrollment, the symptom scale scores of all ASD children decreased, with statistically significant decreased total score of CARS (Z = -5.479, *P* < 0.001), ADOS total score (Z = -2.020, *P* = 0.043) and SA (Z = -2.382, *P* = 0.017), total score of SRS (Z = -4.167, *P* < 0.001), social awareness (Z = -2.459, *P* = 0.014), social cognition (Z = -3.041, *P* = 0.002), social communication (t = 4.820, *P* < 0.001), and social motivation (Z = -4.250, *P* < 0.001). When compared to that at the time of enrollment, the language scores of the neurodevelopment scale of all ASD children were significantly increased (t = -2.206, *P* = 0.029), but the gross motor scores (t = 2.510, *P* = 0.013) and fine motor scores (t = 3.296, *P* = 0.001) significantly decreased. These results suggest that the core symptoms of autism in ASD children were significantly improved 1 year after the intervention, with significantly improved language development (Additional Table 1).

| **Supplementary Table1**  **Comparison of symptom scale and developmental scale scores in ASD children before and after intervention** | | | | |
| --- | --- | --- | --- | --- |
| **Item** | **Basal time（n=176）** | **12 months（n=176）** | **Test** | ***P*** |
| **CARS total score** | 36.00(32.00-40.50) | 33.00(30.00-38.00) | *Z*=-5.479 | <0.001 |
| ADOS total score | 16.00(12.00-20.00) | 16.00(11.00-19.00) | *Z=-2.020* | 0.043 |
| ADOS comparison score | 6.50(6.00-8.00) | 7.00(6.00-8.00) | *Z=-1.814* | 0.070 |
| SA | 14.50(10.00-17.00) | 13.00(10.00-16.25) | *Z=-2.382* | 0.017 |
| RRB | 2.00(1.00-3.00) | 2.00(1.00-3.00) | *Z=0.466* | 0.641 |
| SRS total score | 93.00(76.00-109.00) | 85.00(62.00-106.00) | *Z*=-4.167 | <0.001 |
| Social awareness | 11.00(10.00-13.00) | 11.00(8.50-13.00) | *Z*=-2.459 | 0.014 |
| Social cognition | 18.00(16.00-21.00) | 16.71±4.90 | *Z*=-3.041 | 0.002 |
| Social communication | 33.63±7.90 | 29.82±10.54 | *t*=4.820 | <0.001 |
| Social motivation | 15.00(11.50-20.00) | 13.00(10.00-17.00) | *Z*=-4.250 | <0.001 |
| Autistic mannerisms | 14.08±6.39 | 12.60±6.52 | *Z*=-1.665 | 0.096 |
| GDS DQ | 69.43±14.35 | 67.07±17.74 | *Z*=-1.832 | 0.067 |
| Adaptive behavior | 66.97±15.99 | 64.00(52.00-80.50) | *t*=0.037 | 0.971 |
| Gross motor | 76.00(66.00-88.00) | 74.98±15.10 | *t*=2.510 | 0.013 |
| Fine motor | 76.87±17.32 | 72.58±16.44 | *t*=3.296 | 0.001 |
| Language | 50.00(36.00-64.00) | 51.00(39.50-71.00) | *t*=-2.206 | 0.029 |
| Personal social behavior | 59.00(47.00-69.50) | 61.00(46.00-75.50) | *Z*=-1.674 | 0.094 |
| SM | 9.00(8.00-10.00) | 9.00(8.00-10.00) | *Z*=-0.011 | 0.991 |

Data was shown as Mean±SD. Paired sample t test or paired samples Wilcoxon signed rank test was used for analysis.

**Environmental risk factors for regressive ASD**

The regressive group exhibited a higher incidence of early postnatal diseases, including neonatal hypoxic ischemic encephalopathy (HIE), encephalitis, febrile convulsion, epilepsy, and pathological jaundice (*P* = 0.019). They also showed a higher rate of medication use from birth to age 3 years, including antibiotics, montelukast sodium, loratadine, halamethasone/triclosan cream, and antiallergy drugs (*P* = 0.012), when compared to the non-regressive group. However, there were no significant differences between the two groups regarding maternal gestational age (*P* = 0.212), abnormalities during pregnancy (such as infection, placental abnormalities, amniotic fluid abnormalities, gestational diabetes mellitus, and gestational hypertension) (*P* = 0.465), exposure history during pregnancy (such as smoking, drinking, drug use, radiation, and pesticides) (*P* = 0.108), medication use history during pregnancy (like antibiotics, montelukast sodium, and antidepressants) (*P* = 0.332), emotional state during pregnancy (*P* = 0.519), birth history (including asphyxia, amniotic fluid inhalation, cyanosis, and intracranial hemorrhage) (*P* = 0.138), and family history (such as intellectual disability, cerebral palsy, autism, schizophrenia, and depression) (*P* = 0.085). Similarly, there were no significant differences between the two groups regarding whether there was only one child in the family (*P* = 0.091) and whether they lived with their parents (P = 0.149) (Additional Table 2).

| **Supplementary Table2**  **Comparison of pregnancy, perinatal and postnatal risk factors between R-ASD and NR-ASD** | | | | |
| --- | --- | --- | --- | --- |
| **items** | **R（n=105）** | **NR（n=265）** | ***χ*^2^** | ***P*** |
| **Maternal gestational age, n(%)**  ≤24  25-34  ≥35  Miss | 28（26.67）  62（59.05）  11（10.48）  4（3.81） | 56（21.13）  162（61.13）  22（8.30）  25（9.43） | 4.503 | 0.212 |
| **Abnormalities in pregnancy, n(%)**  Yes  No  Miss | 61（58.10）  36（34.29）  8（7.62） | 141（53.21）  93（35.09）  31（11.70） | 1.530 | 0.465 |
| **Exposure history during pregnancy, n(%)**  Yes  No  Miss | 47（44.76）  50（47.62）  8（7.62） | 100（37.74）  124（46.79）  41（15.47） | 4.447 | 0.108 |
| **Drug use history during pregnancy, n(%)**  Yes  No  Miss | 10（9.52）  83（79.05）  12（11.43） | 20（7.55）  199（75.09）  46（17.36） | 2.204 | 0.332 |
| **Emotion during pregnancy, n(%)**  Emotional stability  Less emotional instability  Emotional instability  Miss | 46（43.81）  35（33.33）  17（16.19）  7（6.67） | 105（39.62）  84（31.70）  45（16.98）  31（11.70） | 2.267 | 0.519 |
| **Birth history, n(%)**  Yes  No  Miss | 15（14.29）  86（81.90）  4（3.81） | 28（10.57）  212（80.00）  25（9.43） | 3.964 | 0.138 |
| **Early postnatal disease, n(%)**  Yes  No  Miss | 30（28.57）  71（67.62）  4（3.81） | 46（17.36）  194（73.21）  25（9.43） | 7.966 | 0.019 |
| **Drug use history from birth to age 3, n(%)**  Yes  No  Miss | 48（45.71）  12（11.43）  45（42.86） | 79（29.81）  32（12.08）  154（58.11） | 8.822 | 0.012 |
| **Family history, n(%)**  Yes  No  Miss | 13（12.38）  88（83.81）  4（3.81） | 35（13.21）  201（75.85）  29（10.94） | 4.941 | 0.085 |
| **Whether there was only one child in the family, n(%)**  Yes  No  Miss | 53（50.48）  48（45.71）  4（3.81） | 143（53.96）  97（36.60）  25（9.43） | 4.801 | 0.091 |
| **Whether lived with their parents, n(%)**  Yes  No  Miss | 97（92.38）  4（3.81）  4（3.81） | 226（85.28）  14（5.28）  25（9.43） | 3.805 | 0.149 |

Data was shown as number (percentage). Chi-square test was used in the analysis.
